# Polysiloxane/Polystyrene Thermo-Responsive and Self-Healing Polymer Network via Lewis Acid-Lewis Base Pair Formation

**DOI:** 10.3390/molecules23020405

**Published:** 2018-02-13

**Authors:** Fernando Vidal, Huina Lin, Cecilia Morales, Frieder Jäkle

**Affiliations:** Department of Chemistry, Rutgers University-Newark, 73 Warren Street, Newark, NJ 07102, USA; fer.vidal@rutgers.edu (F.V.); hl519@scarletmail.rutgers.edu (H.L.); cecilialmorales@gmail.com (C.M.)

**Keywords:** boron, Lewis acid, transient polymer networks, organoborane, supramolecular polymers, telechelic polymers, organoboron polymers, self-healing

## Abstract

The use of thermo-reversible Lewis Pair (LP) interactions in the formation of transient polymer networks is still greatly underexplored. In this work, we describe the synthesis and characterization of polydimethylsiloxane/polystyrene (PDMS/PS) blends that form dynamic Lewis acid-Lewis base adducts resulting in reversible crosslinks. Linear PS containing 10 mol % of di-2-thienylboryl pendant groups randomly distributed was obtained in a two-step one-pot functionalization reaction from silyl-functionalized PS, while ditelechelic PDMS with pyridyl groups at the chain-termini was directly obtained via thiol-ene “click” chemistry from commercially available vinyl-terminated PDMS. The resulting soft gels, formed after mixing solutions containing the PDMS and PS polymers, behave at room temperature as elastomeric solid-like materials with very high viscosity (47,300 Pa·s). We applied rheological measurements to study the thermal and time dependence of the viscoelastic moduli, and also assessed the reprocessability and self-healing behavior of the dry gel.

## 1. Introduction

The use of non-covalent bonds in the construction of advanced supramolecular networks has brought about an ever-increasing diversity of polymeric elastomers and soft gels [1,2,3,4,5], which possess very promising applications as stimuli-responsive [6,7], mechanochromic [8], self-repairing [9,10,11], shape-memory [12], and controlled-release [13] materials. It is the combination of fixed covalent bonds within the polymer chains and the dynamic nature of transient supramolecular crosslinks that provides these materials with interesting mechanical properties and features of both irreversible thermosets (permanently crosslinked, rubbery, and elastic) and easily reprocessable and recyclable viscoelastic polymer melts [5,14]. Indeed, supramolecular interactions based on hydrogen-bonding [15,16,17,18,19], π-π stacking [20], inclusion complexation [21], metal-ligand [22,23,24], and ionic interactions [25] have been extensively exploited in the reversible crosslinking of polymers.

A relatively much less explored strategy involves Lewis pair (LP) interactions between Lewis acid (LA) and Lewis base (LB) functionalities as a tool for the formation of reversible supramolecular polymers and networks. As in the case of other non-covalent bonds, an equilibrium is established between the free Lewis acids/bases and the LP adducts. The nature of this equilibrium is determined by the relative LA and LB strength, which in turn depends on both steric and electronic effects, and can be shifted by changes in temperature and concentration [26,27]. The facile functionalization of molecular and polymeric materials with borane Lewis acid groups makes boron-containing building blocks particularly attractive [28,29,30,31,32]. For instance, the group of Severin investigated LB-functionalized boronate esters, in particular catecholboranes, in the supramolecular self-assembly of extended networks from small molecule precursors [33,34]. However, the resulting organogels require the use of solvents and are relatively weak materials due to the lack of true polymeric viscous flow [35,36]. More recently, Brook and coworkers mixed two complementary polydimethylsiloxane (PDMS) polymers, one containing aryl-boronate esters as a LA and the other primary alkyl amines as a LB, to form the first example of thermoreversible crosslinked silicones via non-covalent LP interactions [37]. The formation of materials with alternatively low viscosity, elastomeric, and brittle characteristics was accomplished by relatively small changes in the crosslinking density. Finally, Shaver and coworkers elegantly used non-interacting arylborane- and dimesitylphosphine-bearing polystyrene (PS) to generate thermoresponsive and self-healing organogels by adding dynamic steric “frustration” to the LP interactions and forcing the transient network to form by the activation of a small molecule [38].

**Figure 1:**
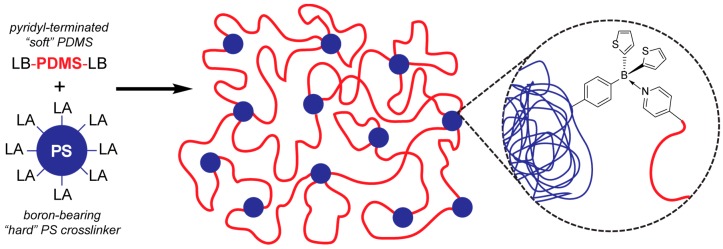
Schematic representation of the proposed crosslinked supramolecular network in the polystyrene/polydimethylsiloxane (PS/PDMS) blend promoted by Lewis acid/Lewis base pairs (LA = Lewis acid, LB = Lewis base).

In this study, we explored whether LP interactions placed at the interface of two immiscible, but complementary polymers—one carrying the LA, the other carrying the LB—could be exploited to render a homogeneously dispersed and thermoreversible supramolecular polymer network (Figure 1). This strategy would obviate the need for external compatibilizers to stabilize the dispersion, such as block-copolymers additives [39]. Ditelechelic PDMS functionalized with Lewis basic pyridine groups at the chain termini provides flexible and low-melting bridging strands required for the viscous flow of the gel at room temperature. A PS random copolymer containing pendant Lewis acidic organoborane moieties in turn serves as a multifunctional crosslinker. Our investigations reveal a dramatic impact of the reversible Lewis acid-base interactions (B-N bond formation) on the transient polymer network thus formed, which exhibits elastomeric, thermo-responsive, and self-healing characteristics.

## 2. Results and Discussion

### 2.1. Synthesis of LA/LB-Containing Polymers and Adduct Formation

**Scheme 1:**
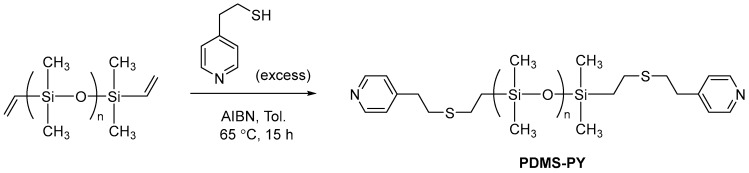
One-step synthesis of pyridine-bearing telechelic PDMS (PDMS-PY) from commercially available vinyl-terminated PDMS.

**Figure 2:**
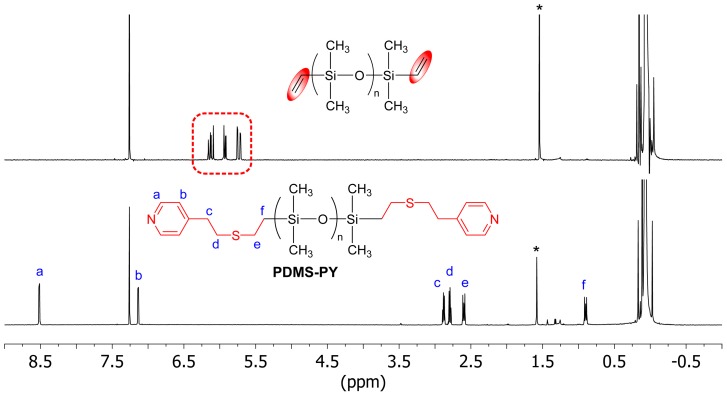
^1^H NMR spectra comparison (25 °C, CDCl_3_) of a vinyl-terminated telechelic PDMS commercial sample (top), and the sample after the installation of the pyridyl end-group (PDMS-PY, bottom) via a “click” reaction. (* = H_2_O trace from “wet” CDCl_3_).

The synthesis of linear soft polymers carrying the Lewis base pyridine (PDMS-PY) at the chain termini was achieved by the thiol-ene “click” reaction between commercially available vinyl-terminated polydimethylsiloxane (degree of polymerization (DP) ~ 67, determined by ^1^H NMR) and pyridylethyl mercaptan (Scheme 1). Complete olefin conversion was achieved with only a moderate excess of the mercaptan (three equivalents per vinyl group), as shown by the total disappearance of the –CH=CH_2_ resonances (*dd* centered at *δ* = 6.12, 5.93, and 5.73 ppm) in the ^1^H NMR spectrum (Figure 2). Formation of new thioether bonds was quantitative in the presence of 2,2′-azobis(2-methylpropionitrile) (AIBN) in toluene at 65 °C for 15 h, also confirmed by the new aromatic signals (doublets centered at *δ* = 8.52, and 7.14 ppm for PDMS-PY) and ethylene signals (centered at *δ* = 2.88, 2.79, 2.60, and 0.90 ppm for PDMS-PY) in the ^1^H NMR of the isolated polymer (Figure 2). A single precipitation in methanol was sufficient to remove all excess reagents from the isolated product. The final LB-content as established by the ^1^H NMR end-group analysis was 0.8 mol % for PDMS-PY, which is somewhat lower than the vinyl content (1.5 mol %) in the parent PDMS. Moreover, gel permeation chromatography (GPC) analysis of the isolated polymer showed a significant increase in the polymer number average molecular weight (*M*_n_) from 5400 Da of the commercial sample to 9600 Da of the pyridyl-functionalized PDMS, which is more than what could be accounted for with only the newly added end-groups (Figure S1a). These discrepancies, together with a decrease in the molecular weight dispersity (*Ð*) from 1.74 to 1.26 and the less than quantitative recovery, can be explained by the loss of a small soluble fraction of PDMS oligomers during the MeOH washing step. Finally, MALDI-TOF analysis of a low molecular weight sample of PDMS-PY (DP ~ 12) obtained under otherwise identical conditions further supports the structure of the telechelic polymers (Figure S2). First, the difference between peaks (Δ = 74.02 *m*/*z*) corresponds to the mass of the PDMS repeating unit (–SiMe_2_O–), and second, the experimental peak at *m*/*z* = 1226.62 and the isotopic distribution simulated for a molecular formula of C_42_H_96_O_11_Si_12_N_2_S_2_Na (the sum of the masses for 11 repeat units, the chain-end groups, and Na) matched with an error of less than 4.5 ppm.

**Scheme 2:**
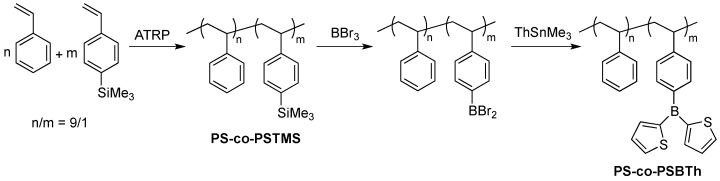
Synthesis of linear PS-based random copolymer containing 10 mol % of Lewis acidic organoboron moieties with pendant thienyl groups (PS-co-PSBTh).

We employed atom-transfer radical polymerization (ATRP) conditions for the controlled and large-scale synthesis of the polystyrene-based random copolymer precursor, PS-co-PSTMS, containing 10 mol % of –SiMe_3_ pendant groups (Scheme 2). The ratio of PS to PS-TMS in the isolated product (n:m) matched well the monomer feed ratio, as determined by ^1^H NMR (see experimental description). The selective exchange of the trimethylsilyl groups with BBr_3_ and subsequent substitution of Br with thiophene groups was achieved by following efficient procedures previously developed in our group [40,41]. Thus, linear polystyrene, PS-co-PSBTh, containing 10 mol % of randomly distributed di-2-thienylboryl pendant groups was conveniently obtained in a high yield (81%) via a two-step, one-pot reaction from the TMS-functionalized precursor. ^1^H NMR analysis of the isolated polymer product showed the complete conversion of the TMS groups (disappearance of the –SiMe_3_ signal at 0.31 ppm, Figure S3), while new resonances indicated the formation of the substituted organoboron moieties (7.97, 7.81, 7.40 ppm for the dithienylborane groups in PS-co-PSBTh, Figure S5). Peak integrations were consistent with a 10 mol % boron content in the final product, as described in detail in the experimental section and Supporting Information (SI).

**Figure 3:**
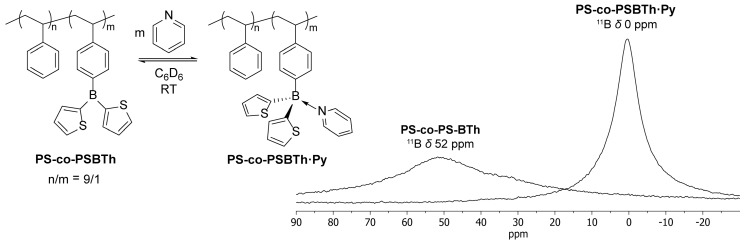
Stoichiometric reaction between PS-co-PS-BTh and pyridine, and the resulting ^11^B NMR spectra.

The ^11^B NMR spectrum of the isolated PS-co-PSBTh showed a single broad peak centered at 52 ppm, which is characteristic of an intact tri-coordinated boron center (Figure 3). To study the formation of LA/LB pairs between the highly Lewis acidic pendant boron groups and a Lewis base, a stoichiometric reaction between one equivalent of pyridine and PS-co-PSBTh was performed. The ^11^B NMR of the resulting mixture showed a dramatic upfield shift to 0 ppm (Δ*δ* = 52 ppm), indicating that the equilibrium in solution is mostly pushed to the formation of the LA·LB adduct. Indeed, the addition of an excess five equivalents of pyridine did not appreciably change the chemical shift, further demonstrating that the boron is vastly tetra-coordinated with only one equivalent of pyridine. The presence of only 10 mol % of boron centers per PS-chain ensured that the polymer·LB adduct remained soluble in C_6_D_6_, as opposed to a previously characterized PSBTh homopolymer which immediately precipitated [26].

### 2.2. Preparation of PDMS/PS Gel Formulation and Physical Characterization

**Figure 4:**
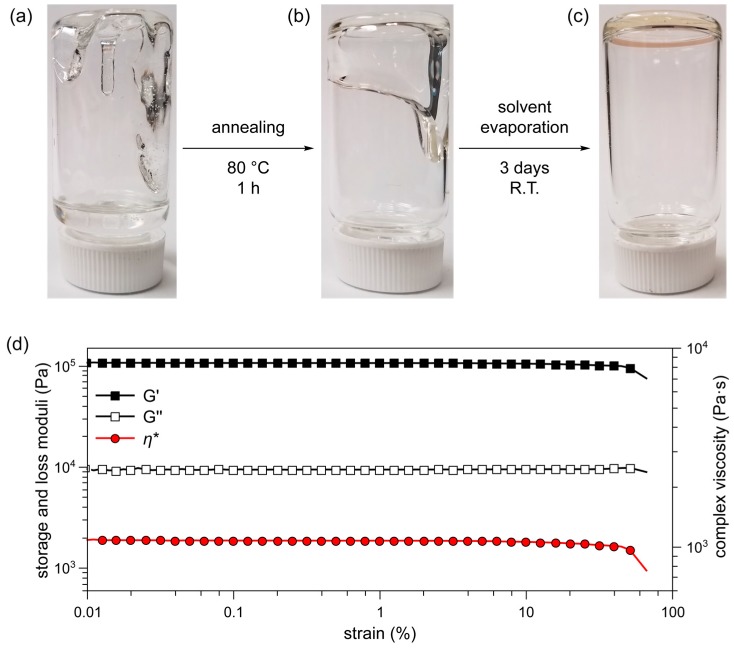
Photographs of PY-PSBTh physical blend: (**a**) soon after the PDMS/PS toluene solutions are mixed (notice the solvent phase separated from the organogels); (**b**) homogeneous and viscous organogel after annealing at 80 °C for 1 h; and (**c**) final PDMS/PS blend after solvent evaporation, PY-PSBTh; (**d**) Strain sweep (100 rad/s at 25 °C) of PY-PSBTh dry gel.

PDMS/PS blends were conveniently prepared at room temperature by mixing solutions of PDMS-PY (~12 wt % in toluene) and PS-co-PSBTh (~5 wt % in CH_2_Cl_2_) in stoichiometric ratios. Upon contact with the PS solution, the PDMS instantaneously underwent crosslinking, which resulted in a heterogeneous gel that separated from the solvent mixture. This clearly suggests that the proposed pyridine-borane adduct formation (Figure 1), also exemplified by the NMR scale reaction (vide supra), readily occurred, imposing a non-ordered structure with a high concentration of crosslinking points. However, the dynamic nature of the Lewis pair bond should allow for reorganization of the network to a more favorable thermodynamic structure upon disassociation/association of the pyridine-boron bonds. Indeed, the disordered network spontaneously relaxed in a matter of minutes at room temperature and produced a viscous transparent organogel that could flow (Figure 4a). Moreover, heating the mixture at 80 °C for one hour generated a perfectly homogeneous and transparent viscous organogel (note that the viscosity changed with the solvent content), enabled by the thermal reversibility of the crosslinks and rearrangement of the PDMS/PS network (Figure 4b). Finally, the volatiles were allowed to slowly evaporate undisturbed over a period of three days, yielding a transparent and almost colorless rubbery gel, PY-PSBTh (Figure 4c), that would not flow after reversing the scintillation vial. In sharp contrast, a control experiment in which PDMS-PY was mixed with PS-co-PSTMS, containing no boron centers, under identical conditions yielded a heterogeneous mixture of liquid PDMS macroscopically separated from white portions of PS as a consequence of the well-known immiscibility of the two polymers [39,42,43,44]. The drastic difference in polymer miscibility highlights the importance of the LA/LB pairs to ensure the homogeneous dispersion of PS, which accounts for 11 wt % of the total gel mass, in the PDMS domains.

The solid-like nature of the PY-PSBTh xerogel at room temperature was confirmed by oscillatory rheological measurements. A strain sweep experiment at a frequency of 100 rad/s showed that the storage modulus (elastic component, G′) is over one order of magnitude larger than the loss modulus (viscous component, G′′) at all of the strains tested before breaking (Figure 4d). Thus, the G′ > G′′ in the linear viscoelastic region indicates that the internal structure at room temperature is in fact that of a highly crosslinked elastomer, which can resist strains greater than 10% even at such high frequencies. Moreover, the zero-shear viscosity (*η*_0_), determined as the complex viscosity (η*) at low shear-rates (Figure S7), is much higher (47,300 Pa·s) compared to that of the free-flowing parent vinyl-terminated PDMS (0.1 Pa·s). Remarkably, the molecular weight of the PDMS-PY employed in this study (*M*_n_ = 9620 Da) was well below the critical molecular weight of PDMS (reported at *M*_c_ = 34,500 Da, approximate DP > 400, [45]); therefore, we can postulate that the rheological observations are not due to the entanglement of the linear PDMS chains, but mostly due to the reversible crosslinks between the PDMS/PS in the form of Lewis pairs.

**Figure 5:**
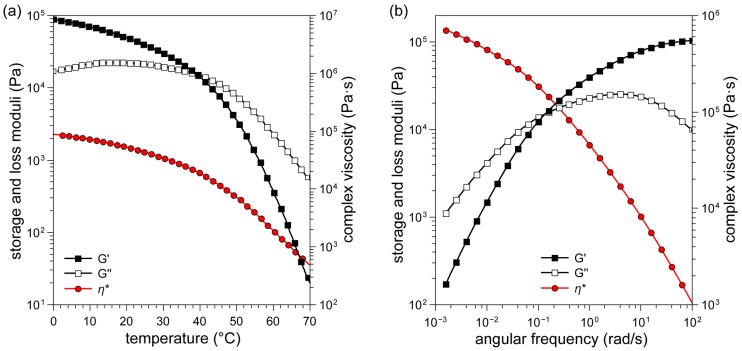
Logarithmic plots of storage modulus (G′, black squares), loss modulus (G′′, white squares), and complex viscosity (η*, red circles) of PY-PSBTh dry gel versus (**a**) temperature (1 °C/min on heating, 1% strain, and 1 rad/s); and (**b**) angular frequency (25 °C, 1% strain).

Next, the thermal reversibility of the B-N crosslinks in the PY-PSBTh blends, already observed qualitatively during the annealing of the organogels, was more accurately studied by heating the solvent-free gel samples at a constant ramp rate (1 °C/min), frequency (1 rad/s), and strain amplitude (1%). The obtained oscillatory rheological data was plotted versus temperature (Figure 5a). The results showed a marked decrease in G′ from 0 °C to 70 °C, consistent with a visual softening of the gels, until it reached a crossover temperature (*T*_cross_ = 39.7 °C) at which G′ = G′′. As we continued heating the sample past the *T*_cross_, the rheological response was dominated by the viscous modulus and the gel was able to flow (G′ < G′′). Importantly, a subsequent cooling cycle overlapped well with the heating ramp and the gel could recover previous values of the viscoelastic moduli at low temperatures, effectively regaining its solid-like behavior (Figure S8). Worth noting here is that the rheological experiments were conducted while exposed to air, thus also demonstrating the good stability of the organoborane moieties to high temperatures and oxygen in the presence of the pyridine end-groups.

Another important feature of transient networks composed of reversible bonds is the time-dependence of the elastic and viscous moduli with respect to the frequency of the applied stress. Thus, we performed dynamic frequency sweeps at 25 °C and the result of the viscoelastic parameters were plotted versus angular frequency (Figure 5b). As expected, both the elastic and viscous moduli changed with frequency, and underwent crossover (G′ = G′′) at 0.144 rad/s. This result clearly highlights the reversibility of the crosslinked network: while the gel behaved like an elastic solid at higher frequencies, i.e., the stresses were applied faster than the time needed for the reorganization of the crosslinks, the gel could flow at lower frequencies since the breaking/formation of the B-N bonds occurred on a similar time-scale as the stress. Moreover, the crossover frequency, *ω*_c_, is directly related to the relaxation time, *τ*, of the viscoelastic network, where *τ* = 2π/*ω*_c_. Thus, the relaxation time of PY-PSBTh, i.e., the average time required to relax by the elastic nodes of the network, was calculated to be 44 s at room temperature.

Thermal characterization of PY-PSBTh by differential scanning calorimetry (DSC) only evidenced a prominent melting transition at −48 °C, which can be assigned to the PDMS domains (Figure S9). In contrast, a glass transition for the PS domains (*T*_g_ of parent PS-co-PSBTh = 118.3 °C) was not detected, nor could any other thermal transition be associated with a crosslinking/decrosslinking reaction, possibly due to the relatively small number of Lewis acid-base pairs. Finally, the thermal stability of the gel, as established by thermal gravimetric analysis (TGA), can be correlated with the mass content of its component: it showed a two-step decomposition profile, with a first onset decomposition temperature, *T*_onset,1_, at 363 °C that accounted for the weight percent of the PS-domains, and a second decomposition temperature, *T*_onset,2_, at 480 °C corresponding to the PDMS-domains (Figure S10a). The maxima of the decomposition steps in the first derivative trace overlapped well with the decomposition maxima of the parent polymers (Figure S10b), hence supporting this assignment.

### 2.3. Observation of Self-Healing Properties

**Figure 6:**
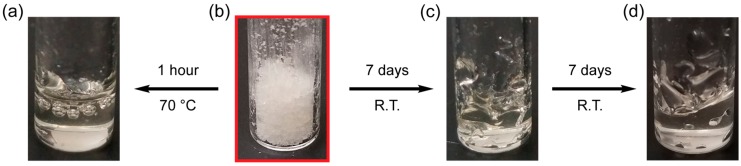
Photographs of solid PY-PSBTh broken xerogel (**b**) and reconstituted PY-PSBTh after standing at room temperature for 1 week (**c**), 2 weeks (**d**), and heating at 70 °C for 1 h (**a**).

The easy access to the flowing regimes and relative short relaxation times at room temperature for PY-PSBTh suggest that this material should be suitable for reprocessability and may display self-healing properties. Indeed, a sample of pulverized PY-PSBTh that was allowed to rest undisturbed at room temperature and under no stress, except for that from gravity, was able to almost completely recover the bulk shape within a week (Figure 6). Even larger gaps were “healed” after standing for an extra week, and the gel almost occupied the volume of the vial. Clearly, by allowing the disassociation of the crosslinks, the PDMS chains could slowly flow so that the soft domains could blend together through the ruptured pieces and reform the network structure. This process could be accelerated at 70 °C, where it took about 1 h to rebuild the network by effectively melting the sample. This is another indication of the transient nature of the B-N crosslinks, which otherwise would not allow the reformation of the gel material.

## 3. Materials and Methods

### 3.1. General Considerations

All oxygen- and moisture-sensitive manipulations were carried out under an inert atmosphere using either standard Schlenk techniques on a dual-manifold Schlenk line or a N_2_-filled glovebox. NMR data were acquired at 25 °C on a 500 MHz Bruker AVANCE spectrometer (499.9 MHz ^1^H, 125.7 MHz ^13^C, 160.4 MHz ^11^B, and 470.4 MHz ^19^F) or on a Varian INOVA 600 MHz spectrometer (599.7 MHz ^1^H, 150.8 MHz ^13^C, 192.4 MHz ^11^B, and 564.3 MHz ^19^F). ^11^B NMR spectra were acquired with boron-free quartz NMR tubes either on the Varian INOVA 600 with a boron-free 5-mm dual broadband gradient probe (Nalorac, Varian Inc., Martinez, CA, USA) or the 500 MHz Bruker Auto Avance with a 5-mm PH SEX 500S1 11B-H/F-D probe. ^1^H and ^13^C spectra were referenced internally to solvent signals and all other NMR spectra externally to SiMe_4_ (0 ppm); chemical shifts are reported as parts per million relative to SiMe_4_.

Boron tribromide, *N*,*N*,*N*′,*N*″,*N*″-pentamethyldiethylenetriamine (PMDETA), pyridine, and 2,2′-azobis(2-methylpropionitrile) (AIBN) were purchased from Sigma-Aldrich, , St. Louis, MO, USA. Copper (II) bromide and 2-phenylethyl bromide were purchased from Acros Organics. Vinyl-terminated polydimethylsiloxane (*M*_n_ = 5400 Da, *Ð* = 1.74, 1.5 mol % of vinyl groups) was purchased from Gelest. The following compounds were prepared according to published procedures: 4-trimethylsilylstyrene [46,47,48], 2-trimethylstannylthiophene [49], and 2-(pyridin-4-yl)ethane-1-thiol [50]. 4-Trimethylsilylstyrene and pyridine were dried over CaH_2_, vacuum distilled, and degassed via several freeze-pump-thaw cycles prior to use.

MALDI-TOF MS analyses were performed on a Bruker Ultraflextreme in linear mode. Anthracene (10 mg/mL) was used as the matrix and mixed with the samples (10 mg/mL in CH_2_Cl_2_) in a 1:1 ratio, and then spotted on the wells of a target plate. Red phosphorus was used for calibration. GPC analyses were performed using a Viscotek GPCmax equipped with a VE 2001 GPC solvent/sample module, a 2600 PDA detector, a TDA 305 triple detector array, and a column set consisting of a PLgel 5-mm mixed-D and two PLgel 5-mm mixed-C columns. The system was calibrated against narrow polystyrene standards (10) in the molecular weight range of 580 to 371,100 Da.

### 3.2. Syntheses and Characterizations

#### 3.2.1. Synthesis of Poly(styrene-*co*-4-trimethylsilylstyrene), PS-co-PSTMS

The random copolymer precursor poly(styrene-*co*-4-trimethylsilylstyrene), PS-co-PSTMS, was prepared by the atom transfer radical polymerization (ATRP) of styrene and 4-trimethylsilylstyrene in a 9:1 molar ratio, respectively. This procedure was adapted from that of the corresponding poly-(4-trimethylsilylstyrene) homopolymer [40,41]. Inside a N_2_-filled glovebox, 3.61 g (0.019 mol) of phenyl ethyl bromide, 2.80 g (0.019 mol) of CuBr, 68.80 g (0.39 mol) of freshly distilled 4-trimethylsilylstyrene, and 365.7 g (3.51 mol) of freshly distilled styrene were added to a Schlenk flask and the mixture was diluted with anhydrous anisole (200 mL). The reaction flask was interfaced to a dual-manifold Schlenk line and the solution was degassed with a slow stream of dry N_2_ for 30 min. Then, 3.38 g (0.019 mol) of PMDETA were quickly transferred via syringe. The polymerization mixture was immersed in a 110 °C oil bath and vigorously stirred for 5 h. The reaction was terminated by placing the flask on ice. The mixture was then filtered through a short plug of neutral alumina and the filter was repeatedly washed with tetrahydrofuran (THF). The combined organic phases were concentrated and precipitated in a large excess of MeOH. Reprecipitation of the product into MeOH was repeated three times from the smallest amount of THF needed. Finally, the product was precipitated into hexanes and dried under vacuum at 100 °C overnight to yield a white powder. The ratio of PS to PS-TMS in the product (n:m) was determined by integration of the peaks in the ^1^H NMR for the PS/PS-TMS (overlapping peaks centered at 6.57 ppm for the 2,6-aryl H’s) compared to PS-TMS (centered at 0.31 ppm for the SiMe_3_ exclusively). Yield = 372 g (85%). *M*_n_ = 16.4 kDa, *Ð* = 1.62. n:m = 9:1 (10 mol % in TMS groups). *T*_g_ = 103.4 °C.

^1^H NMR (499.9 MHz, CDCl_3_, 25 °C): *δ* 7.14 (br s, PS *m*,*p*-aryl and PS-TMS 2,6-aryl overlapped), 6.63–6.52 (br s, PS *o*-aryl and PS-TMS 3,5-aryl overlapped), 2.26–1.90 (br, PS and PS-TMS –CH_2_C*H*– overlapped), 1.50 (br, PS and PS-TMS –C*H*_2_CH– overlapped), 0.31 (s, Si*Me*_3_). ^13^C NMR (150.8 MHz, CDCl_3_, 25 °C): 145.8 (br, PS 1-aryl), 137.1, 136.8 (PS-TMS 1-aryl and 4-aryl), 133.2 (br, PS-TMS 3,5-aryl) 128.1 (br, PS and PS-TMS 2,6-aryl, overlapped), 127.8 (br, PS 3,5-aryl), 125.8 (br, PS 4-aryl) 47–42 (br, –CH_2_*C*H–), 40.5 (br, –*C*H_2_CH–), −0.77 (Si*Me*_3_).

#### 3.2.2. Synthesis of Poly(styrene-*co*-4-(di-2-thienylboryl)styrene), PS-co-PSBTh

The one-pot synthesis of polystyrene random copolymer containing 10 mol % of di-2-thienylboryl units, PS-co-PSBTh, was adapted from the synthesis of the corresponding borane homopolymer [40]. Caution: BBr_3_ is toxic and highly corrosive! Inside a N_2_-filled glovebox, a solution of BBr_3_ (0.82 g, 3.27 mmol) in 10 mL of dry CH_2_Cl_2_ was added dropwise at room temperature to a stirring solution of PS-co-PSTMS (3.00 g, 2.69 mmol of TMS groups) in 20 mL of dry CH_2_Cl_2_. After the addition was completed, the solution was stirred at room temperature for 24 h. ^1^H NMR analysis of a reaction aliquot showed the complete replacement of the TMS groups with –BBr_2_. Subsequently, a solution of 2-(trimethylstannyl)thiophene (1.66 g, 6.72 mmol) in 5 mL of dry CH_2_Cl_2_ was added at room temperature, and the reaction mixture was stirred overnight. The reaction solution was concentrated under vacuum to 5 mL, and the polymer was recovered by repeated precipitation from toluene into hexanes. The resulting off-white solid was washed with hexanes and dried under high vacuum at 50 °C overnight to give a white powder. The ratio of PS to PS-BTh in the product (n:m) was determined by integration of the peaks in the ^1^H NMR for PS/PS-BTh (overlapping peaks centered at 7.10 ppm) compared to PS-BTh (centered at 7.40 ppm for the 4-Th H’s). Yield: 2.60 g (79%). *M*_n_ = 19.0 kDa, *Ð* = 1.20. n:m = 9:1 (10 mol % in B-thiophene groups). *T*_g_ = 118.3 °C.

^1^H NMR (499.9 MHz, CDCl_3_, 25 °C): *δ* 7.97 (br s, PS-BTh 5-Th), 7.81 (br s, PS-BTh 3-Th), 7.53 (br s, PS-BTh 2,6-aryl), 7.40 (br s, PS-BTh 4-Th), 7.10 (br s, PS *m*,*p*-aryl and PS-BTh 3,5-aryl overlapped), 6.65 (br s, PS *o*-aryl), 1.93 (br, PS and PS-BTh –CH_2_C*H*– overlapped), 1.49 (br, PS and PS-BTh –C*H*_2_CH– overlapped). ^13^C NMR (150.8 MHz, CDCl_3_, 25 °C): 148.4 (br, PS-BTh 4-aryl), 145.7 (br, PS 1-aryl), 145.2 (PS-BTh 2-Th), 142.5 (PS-BTh 3-Th), 140.6 (br, PS-BTh 1-aryl), 137.1 (PS-BTh 5-Th), 129.0 (PS-BTh 4-Th), 128.1 (br, PS and PS-BTh 2,6-aryl, overlapped), 127.8 (br, PS 3,5-aryl), 126.9 (br, PS-BTh 3,5-aryl), 125.8 (br, PS 4-aryl) 47–42 (br, –CH_2_*C*H–), 40.6 (br, –*C*H_2_CH–). ^11^B NMR (160.4 MHz, C_6_D_6_, 25 °C): *δ* 52 ppm (broad).

#### 3.2.3. Reaction of PS-co-PSBTh with Pyridine

PS-co-PSBTh (45.4 mg, 0.037 mmol of LA) was dissolved in 0.4 mL of C_6_D_6_. To this solution, an equimolar amount of pyridine (2.9 mg, 0.037 mmol) in 0.2 mL of C_6_D_6_ was added at room temperature with a syringe from a stock solution. Both solutions where mixed vigorously, transferred to a quartz (boron-free) NMR tube, and analyzed by NMR.

^1^H NMR (499.9 MHz, C_6_D_6_, 25 °C): *δ* 8.36 (br s, Py), 7.36–6.73 (br s, PS-BTh and PS aryl H’s, overlapped), 6.27 (br s, Py), 2.11 (br, PS and PS-BTh –CH_2_C*H*– overlapped), 1.62 (br, PS and PS-BTh –C*H*_2_CH– overlapped). ^11^B NMR (160.4 MHz, C_6_D_6_, 25 °C): *δ* 0 ppm (broad).

#### 3.2.4. Synthesis of Pyridine-Terminated Telechelic PDMS, PDMS-PY

This procedure was adapted from a previously published method for a “click” side-chain modification of PDMS with 2-(pyridin-4-yl)ethane-1-thiol [50]. Inside a N_2_-filled glovebox, a high-pressure glass vessel containing a magnetic stir bar was loaded with 10.01 g of vinyl-terminated telechelic PDMS (2.02 mmol of vinyl groups) and diluted with 15 mL of toluene. Then, AIBN (198 mg, 1.20 mmol) and 2-(pyridin-4-yl)ethane-1-thiol (1.68 mg, 12.08 mmol) were added, the vessel was tightly closed, and the mixture was stirred at 65 °C in an oil bath for 15 h. After cooling down to room temperature, the reaction mixture was filtered to remove any small particles and slowly precipitated over vigorously stirred MeOH (500 mL). The high molecular weight fraction of the polymer was isolated by centrifugation followed by decanting of the MeOH supernatant, washed with fresh MeOH (2 × 50 mL), and finally dried under high vacuum at 50 °C until no MeOH peaks were detected in the ^1^H NMR spectrum (the lower molecular weight fraction was discarded from the MeOH wash). The isolated pyridine-terminated telechelic PDMS was a colorless oil, soluble in most organic solvents (including hexanes). Yield = 7.05 g (70%). Pyridyl content = 0.8 mol %. *M*_n_ = 9.60 kDa, *Ð* = 1.27. *T*_m_ = −48.1 °C, −38.0 °C.

^1^H NMR (499.9 MHz, CDCl_3_, 25 °C): *δ* 8.51 (d, *J* = 5.0 Hz, py H’s, chain-end), 7.14 (d, *J* = 4.0 Hz, py H’s, chain-end), 2.88 (t, *J* = 6.2 Hz, py–C*H*_2_–, chain-end), 2.79 (m, –C*H*_2_SCH_2_–, chain-end), 2.60 (m, –CH_2_SC*H*_2_–, chain-end), 0.90 (t, *J* = 6.7 Hz, –Si(CH_3_)_2_C*H*_2_–, chain-end), 0.07 (s, –OSi(C*H*_3_)_2_–, main-chain).

#### 3.2.5. Procedure for the Preparation of Gel Formulations

The blends of PDMS/PS polymers were prepared as follows. Inside a N_2_-filled glovebox, a 20-mL scintillation vial was charged with 500 mg of PDMS-PY (0.050 mmol of LB) and dissolved in 4 mL of toluene. Separately, an equimolar amount of PS-co-PSBTh (66 mg, 0.050 mmol of LA) was dissolved in 1 mL of CH_2_Cl_2_. The PS-copolymer solution was quickly transferred via pipette at room temperature, upon which the PDMS solution in toluene immediately gelled. The gel/solvent mixture was capped, vigorously shaken, and placed in a heated bath at 80 °C for 1 h, after which a homogeneous viscous liquid was formed. The vial was equilibrated at room temperature, uncapped, and allowed to slowly evaporate undisturbed at room temperature for three days. At this point, the residual volatiles were removed under vacuum from the obtained gels at room temperature for 2 h.

#### 3.2.6. Rheology Measurements

Oscillatory shear measurements were performed on a Discovery HR-2 rheometer (TA Instruments) with sandblasted 25-mm parallel plates. Gels were loaded onto a thermally regulated Peltier plate with a clean spatula at 70 °C to remove the thermomechanical history of the polymer blends. Axial force was allowed to relax while taking the geometry gap to 400 μm (trim gap of 425 μm). Dynamic tests were performed in the viscoelastic linear region after performing dynamic strain sweeps at 25 °C after 15 min of equilibration (100 rad/s, 0.01–100% strain). Dynamic stress sweeps (10–200,000 μN·m, 100 points per decade) were run at a frequency of 1.0 rad/s to determine the zero-shear viscosity. Dynamic temperature tests were run at a ramp rate of 1 °C/min from 70 °C to 0 °C with a strain of 1% and a frequency of 1.0 rad/s. The crossover temperature was reported from the heating ramp, repeated immediately after the first cooling ramp and with the same experimental parameters. A second cooling ramp from 70 °C to 0 °C was performed with the same experimental parameters. Dynamic frequency sweeps (100 to 0.001 rad/s, 10 points per decade) were recorded at 25 °C after 15 min of equilibration with a strain of 1%. Data analysis was carried out with the TRIOS Software Version 4.2.1 (TA Instruments, New Castle, DE, USA).

## 4. Conclusions

We disclose a novel strategy to construct transient polymer networks by blending together two immiscible linear polymers that are functionalized with complementary Lewis acid and Lewis base groups, rendering perfectly air-stable, homogeneous, and transparent solid-like gels. The Lewis pair formation between highly Lewis acidic organoboron moieties, in the form of 10 mol % of pendant di-2-thienylboryl pendant groups attached to PS chains, and Lewis basic pyridine groups attached at the chain termini of ditelechelic PDMS, provide critical thermo-reversible crosslinking points. The latter impart viscoelastic properties as demonstrated by thermal and rheological characterizations, as well as reprocessability and self-healing qualities. Our results further suggest vast opportunities for tuning the properties of transient networks by judiciously selecting the strength of the Lewis acid/Lewis base interactions and the creation of new thermoplastic elastomers with self-healing properties, a subject we are currently investigating.

## Supplementary Materials

Supporting Figures S1–S10 are available at http://www.mdpi.com/1420-3049/23/2/405/s1.
