# Supplementary material for: Polysiloxane/Polystyrene Thermo-Responsive and Self-Healing Polymer Network via Lewis Acid-Lewis Base Pair Formation
Source: Molecules. 2018 Feb 13;23(2):405. doi: 10.3390/molecules23020405 (PMC6017355; doi:10.3390/molecules23020405)
Supplement: Supplementary file 1 [file molecules-23-00405-s001.pdf]

Supporting Information

Polysiloxane/Polystyrene Thermo-Responsive and Self-Healing Polymer Network via Lewis Acid-Lewis Base Pair Formation

Fernando Vidal, Huina Lin, Cecilia Morales, and Frieder Jäkle*

Department of Chemistry, Rutgers University-Newark, 73 Warren Street, Newark, New Jersey 07102, United States

(A) FIGURES

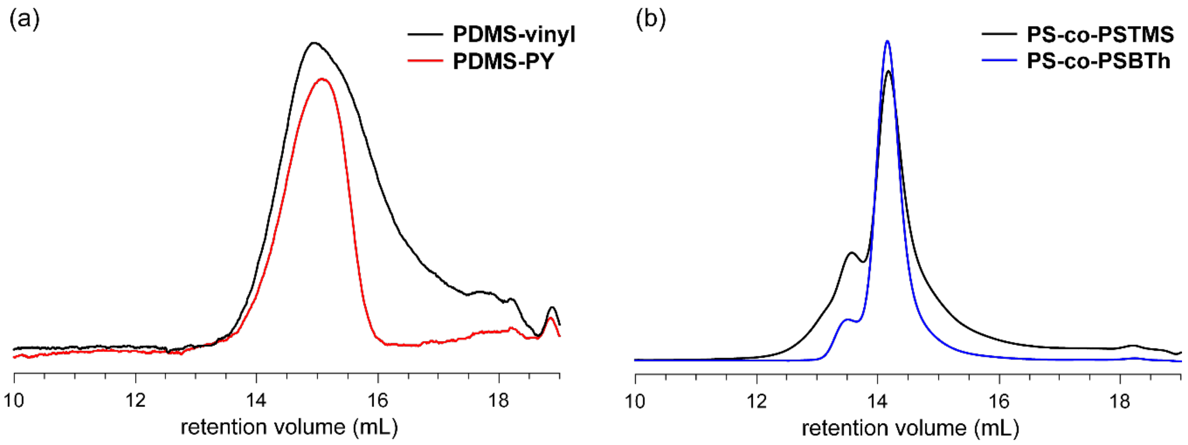

Figure S1. Gel permeation chromatograms of (a) PDMS polymers and (b) PS polymers.

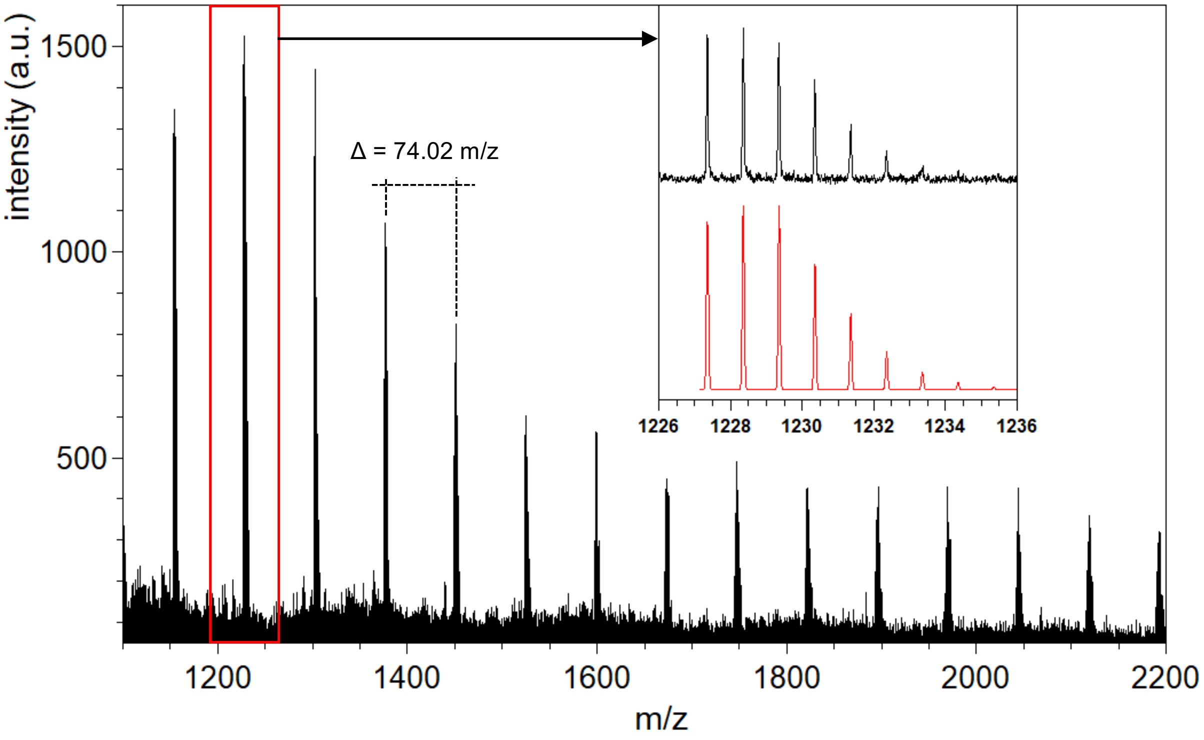

Figure S2. Portion of the MALDI-TOF mass spectrum of a low-molecular weight sample of **PDMS-PY**. Insert: zoom-in at the same experimental spectrum (top) and simulated spectrum for a polymer chain with a molecular formula of $C_{42}H_{96}O_{11}Si_{12}N_2S_2Na$ (bottom).

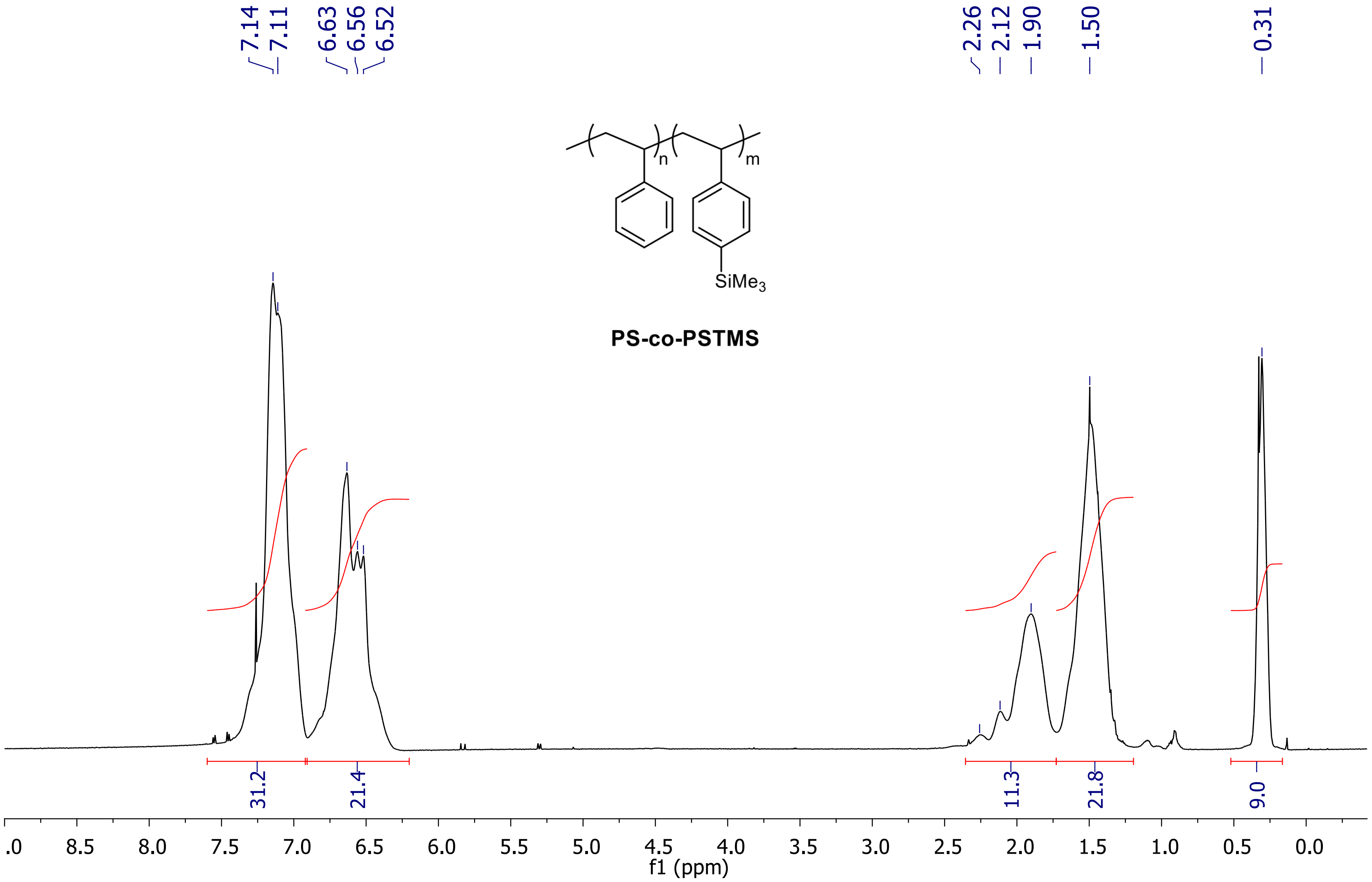

Figure S3. ^{1}H NMR ($CDCl_3$, 25 °C) of isolated **PS-co-PSTMS**.

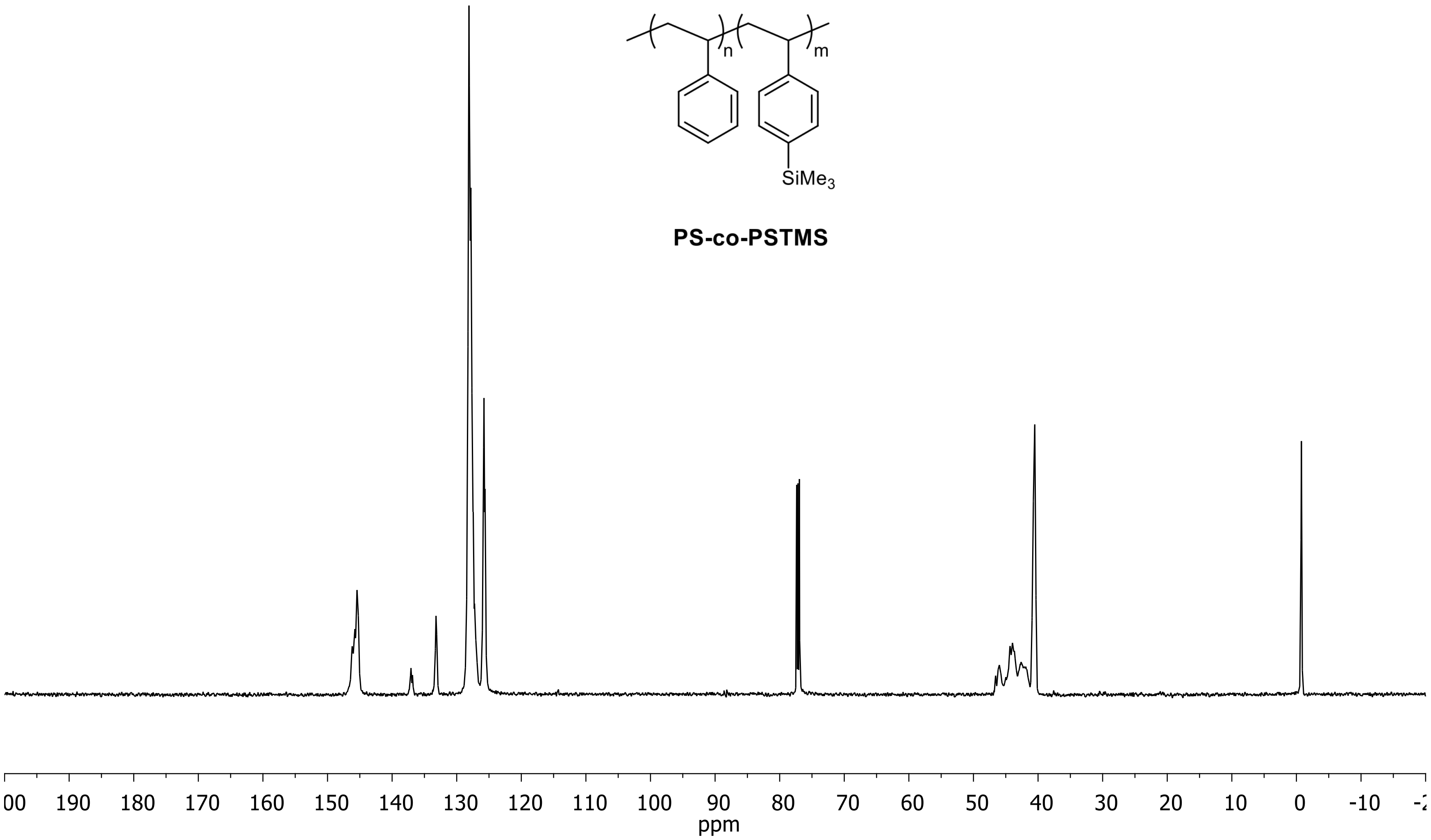

Figure S4. ^{13}C NMR ($CDCl_3$, 25 °C) of isolated **PS-co-PSTMS**.

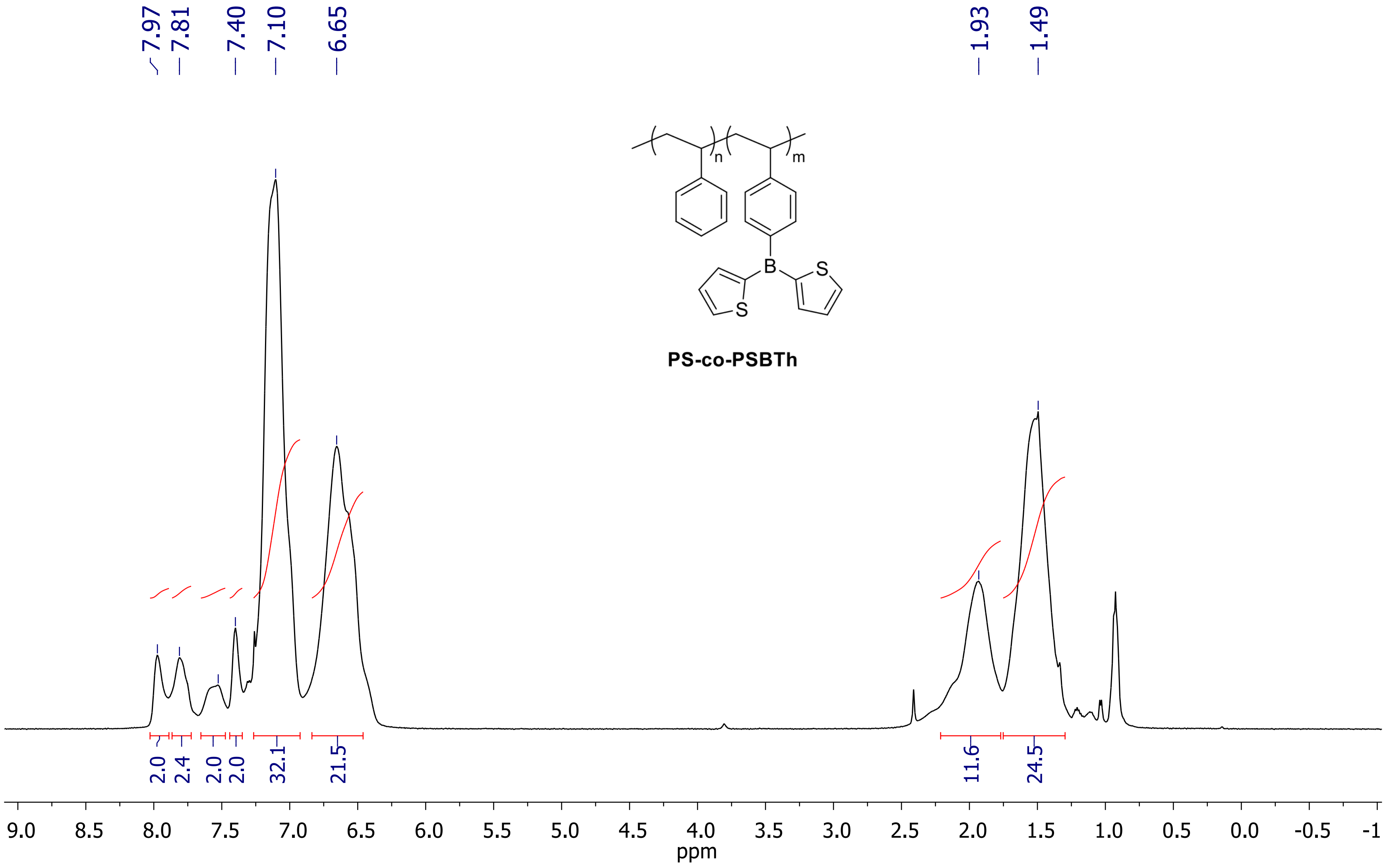

Figure S5. ^{1}H NMR ($CDCl_3$, 25 °C) of isolated **PS-co-PSBTh**.

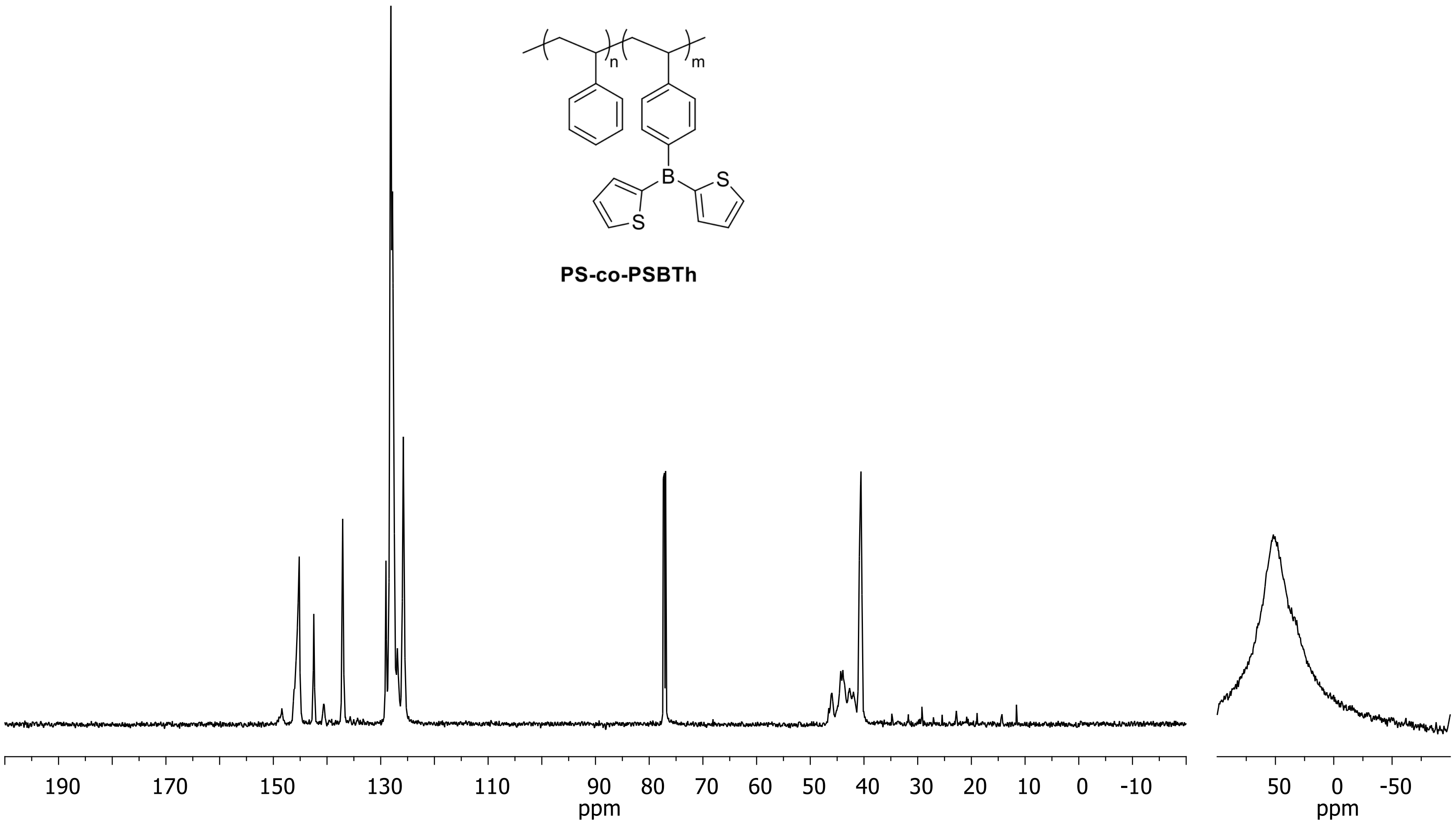

Figure S6. ^{13}C NMR ($CDCl_3$, 25 °C, left) and ^{11}B NMR (C_6D_6, 25 °C, right) of isolated **PS-co-PSBTh**.

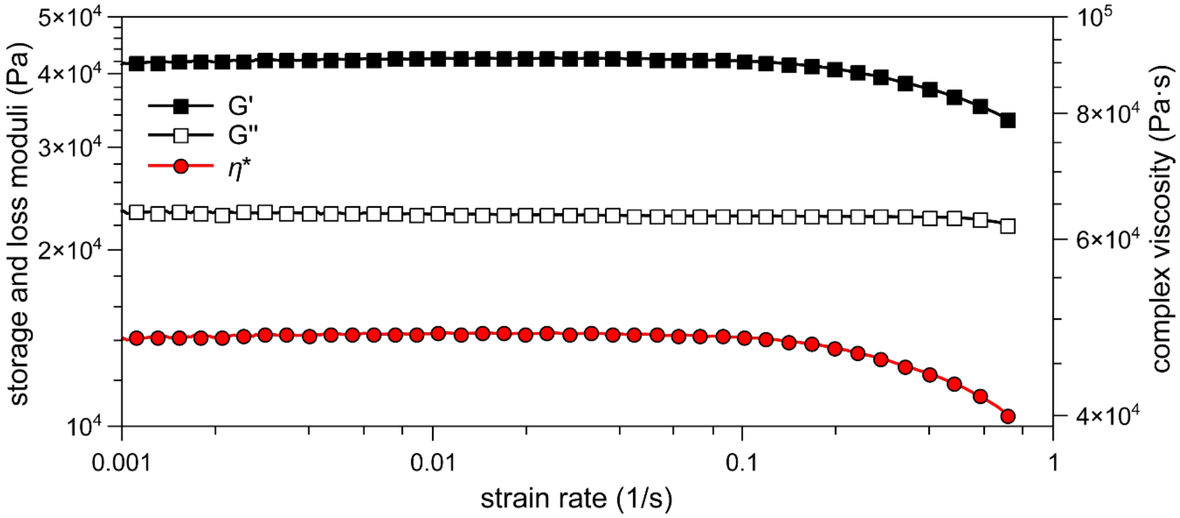

Figure S7. Strain-rate sweep (1 rad/s, at 25 °C) of **PY-PSBTh** dry gel (d).

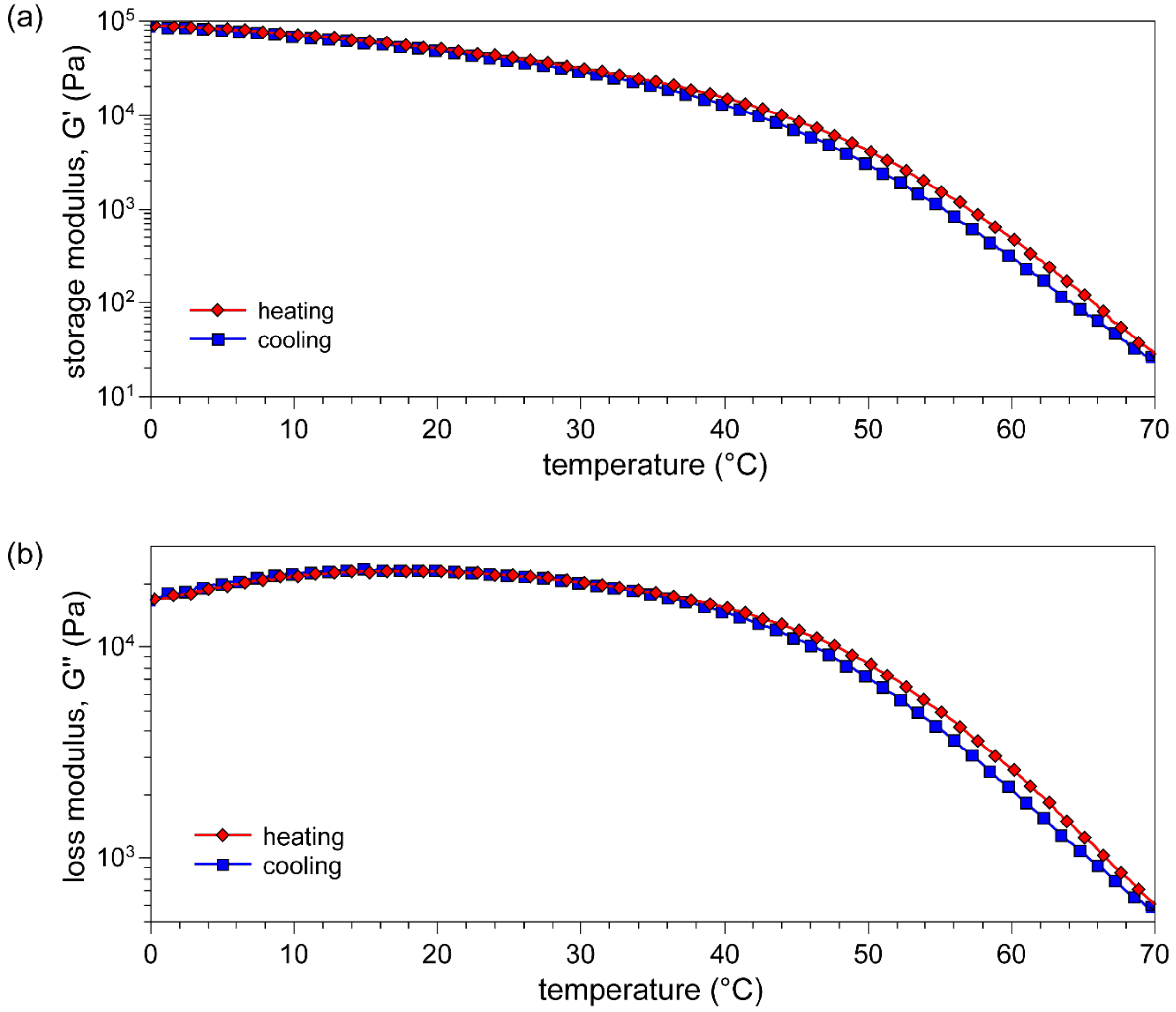

Figure S8. Temperature ramps (1 °C/min, 1 rad/s, 1%) of **PY-PSBTh** dry gel on cooling (blue) and heating (red).

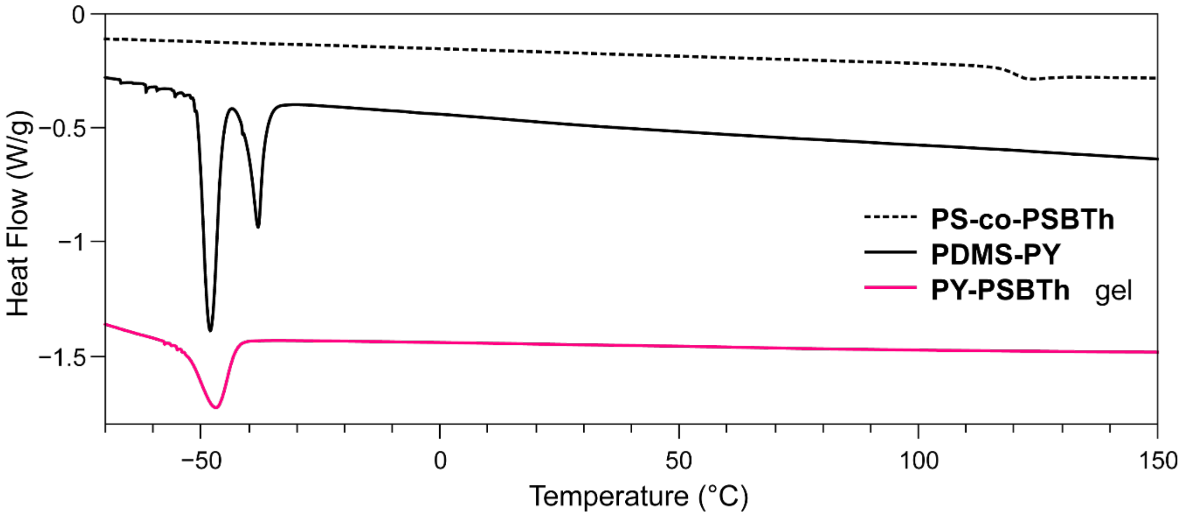

Figure S9. DSC thermograms of isolated **PS-co-PSBTh** (dotted black line), **PDMS-PY** (solid black line), and **PY-PSBTh** blend (solid pink line), ramp rate of 10 °C/min.

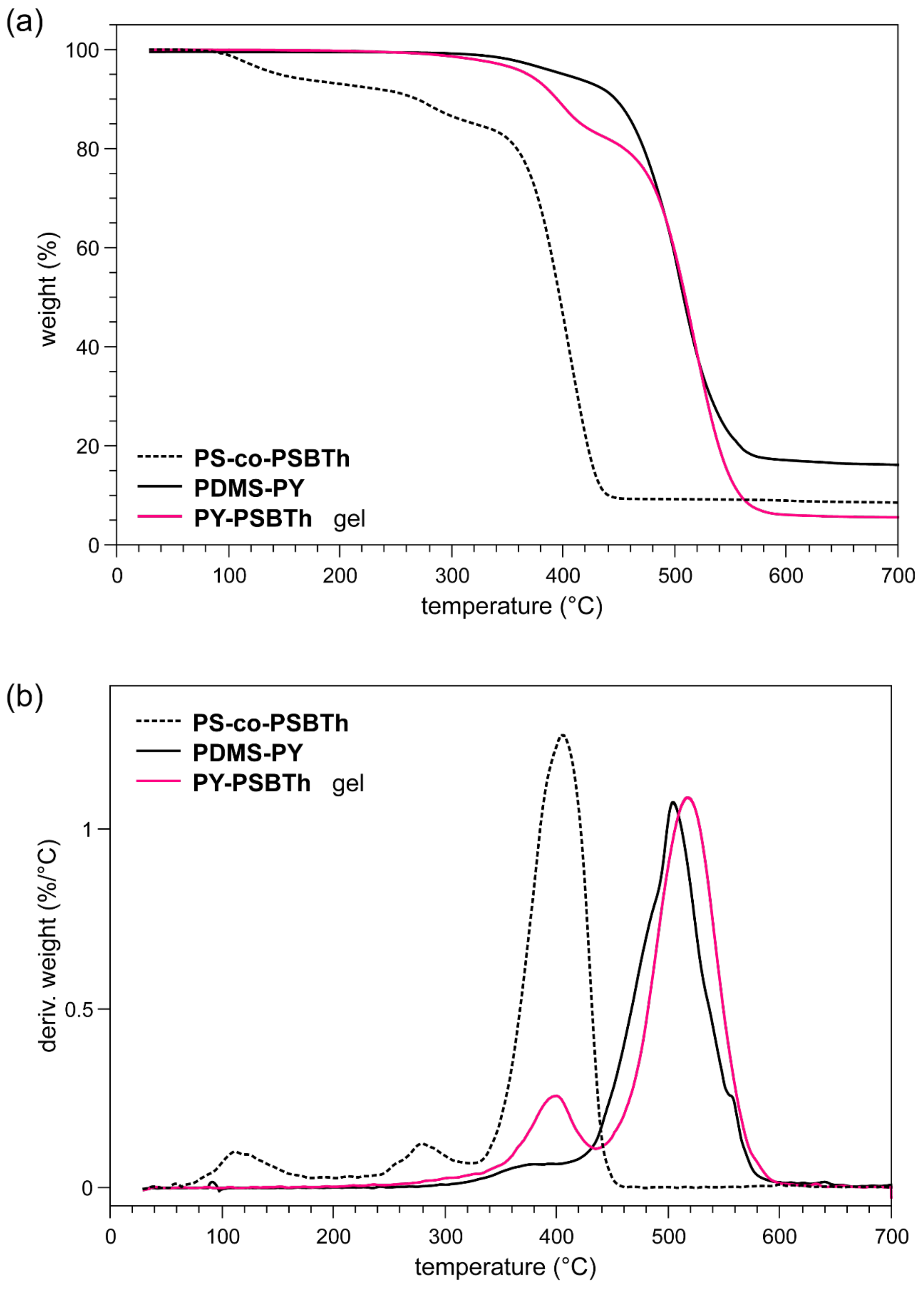

Figure S10. TGA traces of **PS-co-PSBTh** (dotted black line), **PDMS-PY** (solid black line), and **PY-PSBTh** blend (solid pink line), ramp rate of 10 °C/min: (a) weight (wt %) vs temperature (°C); (b) derivative (wt %/°C) vs temperature (°C).
